# Exploratory Factor Analysis of Medical Students’ Perceptions of Medical Cannabis Scale

**DOI:** 10.7759/cureus.25749

**Published:** 2022-06-08

**Authors:** Robin J Jacobs, Michael N Kane

**Affiliations:** 1 Medical and Behavioral Research, Health Informatics, Medical Education, Nova Southeastern University, Fort Lauderdale, USA; 2 Social Work, Florida Atlantic University, Boca Raton, USA

**Keywords:** medical education research, measurement, factor analysis, cannabinoid, medical marijuana, cannabis

## Abstract

Background

There are few published research articles investigating medical students’ perceptions of medical cannabis (MC), including their attitudes toward its efficacy and appropriateness in medicine, concerns for potential adverse effects, and their willingness to prescribe it to patients (in future practice). This research investigated the factor structure of a tool to assess medical students’ perceptions of MC for the purpose of curriculum enhancement.

Methods

Using a voluntary electronic survey, quantitative data were collected between January and March 2022 from 526 medical students enrolled in a large medical school in Florida, United States. A 32-item questionnaire developed by the researchers was used to investigate medical students’ perceptions of MC. The survey was anonymous and took about 10 minutes to complete. Bivariate correlation analyses were conducted prior to performing a principal component analysis with varimax rotation.

Results

Using principal component analysis with varimax rotation, three factors were identified with eigenvalues greater than 1.0 and a cumulative variance of 59.694%. These factors are perceived knowledge of MC, concern for possible adverse effects of MC (e.g., the potential for misuse/dependence), and attitudes toward MC (e.g., cannabis having an acceptable role in medicine, willingness (as a future physician) to help patients access MC, obtaining training about MC in school and residency training, the physician’s role as a prescriber, and efficacy and benefits of MC for certain health conditions).

Conclusions

The development of this kind of brief measure may be valuable for defining the future educational needs of medical students and other health professionals as well as a tool for future research.

## Introduction

Perceptions of medical cannabis (MC) among physicians in training are of particular interest as favorable attitudes toward alternative therapies such as MC among patients are gaining popularity and acceptance [1-9]. Cannabis has been shown to relieve pain and assist in managing certain chronic diseases [10-16] yet may present potential side effects, including dependence [17].

In early 2022, in the United States (USA), 39 states and Washington, DC, legalized MC [18]. Currently, MC is still federally classified as a Schedule I substance, that is, drugs that are not acknowledged for medical use and possess the potential for abuse. In April 2022, the United States House of Representatives passed legislation that attempts to legalize cannabis at the federal level [19]. In this context, early-career physicians will most likely come across patients asking for information on MC’s usefulness and safety as it becomes more socially acceptable [20,21], and legalization continues to expand. In light of these recent events, MC may likely become a significant issue for the healthcare profession and medical trainees in particular who might be expected to inform patients of its efficacy, recommend it, and develop treatment plans. However, it is unclear if medical students have sufficient knowledge about MC or what their perceptions are about its use in medical practice including efficacy and possible adverse effects.

While clinical research on the benefits and adverse effects of MC is still, relatively speaking, in its nascent stages, there is evidence indicating that MC results in some improvement in pain relief, physical functioning, and sleep quality among patients with chronic pain and can help reduce opioid use [22-24]. It has also been suggested that MC can alleviate symptoms related to chemotherapy-induced nausea and vomiting, chronic pain, and multiple sclerosis-related spasticities, among other conditions [25].

In 2021, Weisman and Rodríguez published a systematic review of medical students’ and health professionals’ attitudes toward MC [26]. They found that physicians’ and medical students’ endorsement for legalizing MC has increased from 1991 to 2019 [26]. In addition, 64.4% of the 9,265 medical students from 26 studies believed in MC’s therapeutic utility. Students also reported being concerned about MC’s potential for dependence/addiction and possessed a strong desire for more education about MC while in school [7,26]. Other research studies report that most medical students believe that MC can play a role in the management of several health conditions but resonate with concerns about the risks of MC and may be reticent to recommend it to future patients [27]. Other researchers have reported that prior cannabis use was related to the belief that MC was an effective treatment [8].

As MC increases its social acceptability [20] and laws begin to change in favor of legalization, newly trained physicians will be faced with an increased number of patients looking for treatment options and information on the safety of medical cannabis [21]. To date, however, there are few if any, instruments available that can measure students’ perceptions of MC, including concerns and willingness to use MC in their post-residency practice.

The aim of this study was thus to develop a brief instrument to measure medical students’ perceptions of medical cannabis. Perceived knowledge of MC, concern for potential adverse effects, and attitudes toward legalization and physician prescribing issues were investigated. The development of this kind of tool may be helpful in furnishing the educational needs of medical students and other health professionals. Moreover, the tool may be useful for promoting future research.

## Materials and methods

Sample and questionnaire administration

This study was approved by the Nova Southeastern University Institutional Review Board (protocol number 2022-28). Using an anonymous, electronic survey, quantitative data were collected between January and March 2022 from medical students enrolled in a Florida college of osteopathic medicine via student email listservs. The email contained a letter delineating the purpose of the study and the voluntary nature of the study. The survey was sent at predetermined intervals to encourage participation in completing the survey. The survey took about 10 minutes to complete.

Assessment instrument

The aim of the development of the 32-item questionnaire (created by the researchers) was to evaluate medical students’ attitudes toward MC, concerns for its possible adverse effects, and perceived knowledge about MC. The items included were Likert-type items using a 6-point response set (1=strongly agree, 2=agree, 3=somewhat agree, 4=somewhat disagree, 5=disagree, 6=strongly disagree), many of which were adapted from various research reports [3,27,28-52]. All items were checked for face validity through the agreement of three health professions educators (from medicine, pharmacy, and social work) and three medical students in various years of study. The items assessed for this study were part of a longer instrument that investigated medical students’ attitudes, perceptions, opinions, and knowledge of MC, including legalization and prescribing aspects. In addition, participant demographic data were collected.

Analysis

Out of the 1,447 medical students (class years 1-4) enrolled in the school, 637 students returned the questionnaire cases with less than two-thirds of completed items dropped from the analysis (n=111), leaving 526 completed questionnaires for the final analysis.

Data were analyzed using the Statistical Package for the Social Sciences (SPSS) version 27 (IBM Corp., Armonk, NY, USA) [53]. The researchers visually inspected the observed distributions and conducted tests for skewness and kurtosis (i.e., assessment for normal distributions). Multicollinearity testing was performed (i.e., tolerance and variance inflation factor (VIF), whereby tolerance statistics less than 0.2 and VIF statistics greater than 5.0 indicate multicollinearity); variables were found to be within acceptable VIF limits [54].

Data were also examined to determine if they approximated normal distributions by investigating skewness and kurtosis to evaluate data distributions [55,56]; the data did not deviate from normality to any significant degree.

Bivariate correlation analyses were conducted prior to performing the factor analysis. To reduce the risk of inefficient factor solutions, items that were not statistically significantly correlated with other items were omitted from further analysis [57].

Due to its size (N=526) and lack of multicollinearity, the sample was considered adequate. Nonetheless, to confirm its adequacy, it was assessed using the Kaiser-Meyer-Olkin (KMO) statistic for both individual and multiple variables. It is known that scores for these two analyses may range from 0 to 1, with higher scores more desirable in factor analysis. The KMO statistic for multiple variables was computed at 0.913; scores equal to or greater than 0.9 are considered to be excellent [54]. In addition, statistics for individual scores were computed, with all items ranging from 0.527 to 0.879. Scores above 0.5 are considered acceptable [54]. KMO statistics in this analysis imply that the sample size for the principal component analysis exceeded the minimal requirements.

While various methods of extraction available to factor-analyze data can be used, principal component analysis with varimax rotation was chosen. It is a statistical technique used as an attempt to illuminate the relationship among factors by adjusting the coordinates of data that evolve from the principal component analysis. The adjustment (i.e., rotation) maximizes the variance shared among items. The varimax rotation streamlines the loadings of items by removing the middle ground and identifying the factor upon which data load, resulting in a small number of important salient variables, thus aiding in the interpretation of the results [55]. When examining participants measured on each of the variables, varimax rotation looks for a base that most economically represents each individual. In essence, each person can be adequately described by a linear combination of only a few functions [55].

Principal component analysis

Twenty-two items were analyzed using principal component analysis with varimax rotation in an attempt to reduce the number of correlated items into fewer factors. Eigenvalues, one of the statistics generated in this procedure, were used to identify the variation in the original items that are explained by a particular factor [56]. Eigenvalues less than 1.0 are not considered significant [56].

It is important to note that four items were removed as they did not contribute to a simple factor structure, failing to meet the minimum criterion of having a primary factor loading of 0.5 or above. These items were as follows: 1) “medical cannabis is taught as part of my medical school curriculum,” 2) “I am concerned there is limited evidence of therapeutic benefits from medical cannabis,” 3) “it is acceptable to prescribe medical cannabis by virtual office visits (telehealth),” and 4) “additional research regarding medical cannabis use should be encouraged.” In addition, the item “additional research regarding medical cannabis use should be encouraged” had a floor effect (i.e., there was a lower limit on the survey item, and a large percentage of the participants scored near this lower limit) with 99.2% (N=483) of the students who answered the item reporting that they “agree” with the statement (using combined responses reported under strongly agree, agree, and somewhat agree), resulting in positively skewed data.

## Results

Characteristics of the sample

The age range of the participants was 18-47 years (mean=26 years, SD=3.431). Table 1 shows the sample characteristics.

**Table 1:** Sample Characteristics (N=526)

Characteristic	n	%
Sex		
Female	239	45.4
Male	229	43.5
Preferred not to answer	58	11
Race		
White	330	62.7
Black	12	2.3
Asian or Pacific Islander	96	18.3
Preferred not to answer	88	16.7
Ethnicity		
Hispanic	79	15
Non-Hispanic	330	62.7
Year in medical school		
Year 1 (preclinical)	169	32.1
Year 2 (preclinical)	251	47.7
Year 3 (clinical)	73	13.9
Year 4 (clinical)	33	6.3

Attitudes toward MC

Table 2 reports the frequencies and percentages for the original 22 items. The majority of the participants reported that they agree (combined strongly agree/agree/somewhat agree) that “cannabis has an acceptable role in medicine” (n=513; 97.6%), “there are significant physical health benefits to using MC” (n=454; 86.3%), and “MC helps patients who suffer from chronic, debilitating medical conditions” (n=420; 79.9%). Most participants also felt that “physicians should be able to legally prescribe cannabis as medical therapy” (n=476; 96%), “physicians should recommend medical cannabis as medical therapy” (n=476; 96%), and “cannabis should be reclassified so that it is no longer a Schedule I drug” (n=432; 88.7%).

**Table 2:** Frequencies and Percentages for Survey Items

Items	Strongly agree		Agree		Somewhat agree		Somewhat disagree		Disagree		Strongly disagree	
	Count	Row valid N %	Count	Row valid N %	Count	Row valid N %	Count	Row valid N %	Count	Row valid N %	Count	Row valid N %
I am familiar with the possible therapeutic effects of medical cannabis.	94	23.5%	195	48.8%	81	20.3%	22	5.5%	5	1.3%	3	0.8%
Medical cannabis helps patients who suffer from chronic, debilitating medical conditions.	215	40.9%	205	39%	93	17.7%	5	1%	6	1.1%	2	0.4%
Using cannabis poses serious physical health risks.	12	2.3%	46	8.7%	99	18.8%	157	29.8%	150	28.5%	62	11.8%
Using cannabis poses serious mental health risks.	27	5.1%	59	11.2%	154	29.3%	143	27.2%	101	19.2%	42	8%
Cannabis has an acceptable role in medicine.	159	30.2%	186	35.4%	149	28.3%	21	4%	9	1.7%	2	0.4%
There are significant physical health benefits to using medical cannabis.	127	24.1%	172	32.7%	155	29.5%	56	10.6%	13	2.5%	3	0.6%
I have substantial knowledge about medical cannabis.	39	7.4%	105	20%	153	29.1%	103	19.6%	90	17.1%	35	6.7%
I am extremely confident regarding my current knowledge of medical cannabis.	33	6.3%	79	15%	136	25.9%	106	20.2%	107	20.3%	65	12.4%
I have good knowledge of the side effects of medicinal cannabis.	56	10.6%	129	24.5%	138	26.2%	103	19.6%	72	13.7%	28	5.3%
Physicians should be able to legally prescribe cannabis as medical therapy.	174	35.1%	187	37.7%	115	23.2%	13	2.6%	4	0.8%	3	0.6%
Physicians should recommend medical cannabis as medical therapy.	109	22%	148	29.8%	190	38.3%	39	7.9%	6	1.2%	4	0.8%
As a healthcare provider (in the future), I would be willing to help patients access medical cannabis.	137	27.6%	178	35.9%	135	27.2%	31	6.3%	9	1.8%	6	1.2%
Training about medical cannabis should be incorporated into medical/health/social well-being-related academic (preclinical) curricula.	181	36.5%	185	37.3%	107	21.6%	17	3.4%	3	0.6%	3	0.6%
Training about medical cannabis should be incorporated into residency/field practice (clinical) requirements.	164	33.1%	177	35.7%	126	25.4%	16	3.2%	9	1.8%	4	0.8%
Medical cannabis use can be addictive.	53	10.9%	143	29.4%	165	33.9%	80	16.4%	32	6.6%	14	2.9%
I am concerned with medical cannabis’ potential for abuse/misuse.	67	13.8%	139	28.5%	119	24.4%	85	17.5%	50	10.3%	27	5.5%
I am concerned about the potential side effects of medical cannabis use.	52	10.7%	127	26.1%	132	27.1%	79	16.2%	68	14%	29	6%
Cannabis should be reclassified so that it is no longer a Schedule I drug.	265	54.4%	99	20.3%	68	14%	37	7.6%	13	2.7%	5	1%
Medical cannabis is taught as part of my medical school curriculum.	21	4.2%	29	5.8%	62	12.5%	115	23.2%	172	34.7%	97	19.6%
I am concerned that there is limited evidence of the therapeutic benefits of medical cannabis.	28	5.3%	81	15.4%	147	27.9%	106	20.2%	102	19.4%	62	11.8%
It is acceptable to prescribe medical cannabis by virtual office visits (telehealth).	43	8.8%	81	16.6%	126	25.9%	126	25.9%	83	17%	28	5.7%
Additional research regarding medical cannabis use should be encouraged.	312	64.1%	133	27.3%	38	7.8%	3	0.6%	1	0.2%	0	0%

Concern for possible adverse effects

While less than one-half of the participants reported that they agreed that “using cannabis poses serious mental health risks” (n=240; 45.6%) and even fewer believed that “using cannabis poses serious physical health risks” (n=157; 29.8%), a large proportion believed that MC use can be addictive (n=361; n=74.2%) and were “concerned for MC’s potential for abuse/misuse” (n=325; 66.7%) and potential side effects (n=311; 63.6%).

Perceived knowledge

Only about one-half of the participants (n=297; 56.5%) agreed that they had substantial knowledge about MC. Fewer participants (n=248; 47.2%) felt “extremely confident regarding their current knowledge of MC,” yet 61% (n=323) believed that they “have good knowledge of the side effects of MC.” Only 22.5% (n=112) of participants reported that MC was taught as part of their medical school curriculum.

Factor analysis

Using principal component analysis with varimax rotation, 18 of 22 items were reduced to a three-factor solution. Table 3 depicts the rotated solution with factor loadings.

**Table 3:** Rotated Component Matrix Note: All items were adapted from a variety of previous studies [3,27,28-52].

Items (N=18)	Factor loading
Factor 1	
As a healthcare provider (in the future), I would be willing to help patients access medical cannabis.	0.745
Training about medical cannabis should be incorporated into residency/field practice (clinical) requirements.	0.745
Training about medical cannabis should be incorporated into medical/health/social well-being-related academic (preclinical) curricula.	0.744
Physicians should be able to legally prescribe cannabis as medical therapy.	0.721
Physicians should recommend medical cannabis as medical therapy.	0.706
Cannabis has an acceptable role in medicine.	0.636
There are significant physical health benefits to using medical cannabis.	0.593
Medical cannabis helps patients who suffer from chronic, debilitating medical conditions.	0.579
Cannabis should be reclassified so that it is no longer a Schedule I drug.	0.566
Factor 2	
I am concerned with medical cannabis’ potential for abuse/misuse.	0.823
I am concerned about the potential side effects of medical cannabis use.	0.817
Using cannabis poses serious mental health risks.	0.769
Using cannabis poses serious physical health risks.	0.722
Medical cannabis use can be addictive.	0.688
Factor 3	
I am extremely confident regarding my current knowledge of medical cannabis.	0.879
I have substantial knowledge about medical cannabis.	0.857
I have good knowledge of the side effects of medical cannabis.	0.784
I am familiar with the possible therapeutic effects of medical cannabis.	0.527

**Figure 1:**
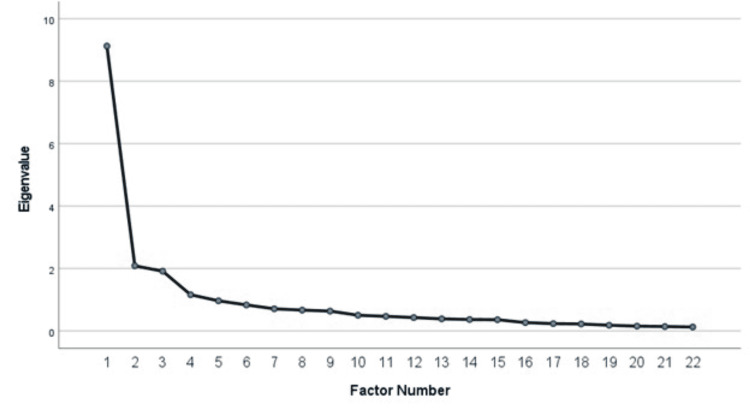
Scree Plot

The items not included in Table 3 for failure to contribute to a simple factor structure are as follows: “medical cannabis is taught as part of my medical school curriculum,” “I am concerned that there is limited evidence of the therapeutic benefits of medical cannabis,” “it is acceptable to prescribe medical cannabis by virtual office visits (telehealth),” and “additional research regarding medical cannabis use should be encouraged.” All factors had an eigenvalue greater than 1.0, and the cumulative variance of 59.694% was calculated. A scree plot was used to confirm the solution (i.e., determine the number of factors to keep in the exploratory factor analysis) (Figure 1).

The three factors included are as follows: 1) attitudes toward MC, 2) concern for possible adverse effects of MC, and 3) perceived knowledge of MC. Factor 1 (perceived knowledge of MC) accounted for approximately 41.5% of the total variance, with nine items. The reliability estimate (Cronbach’s α) for the first factor was calculated at 0.91. The second factor, with five items, accounted for 9.5% of the variance and a reliability estimate of 0.89. Factor 3 accounted for 8.7% of the variance, with four items. The reliability estimate was calculated at 0.89 for the third factor. Table 4 reports the information regarding each factor’s variance and eigenvalue.

**Table 4:** Eigenvalues and Variance of the Factors

Factor	Eigenvalue	Percent of variance	Cumulative percent of the variance
1	9.127	41.486	41.486
2	2.090	9.498	50.985
3	1.916	8.709	59.694

## Discussion

Using principal component analysis with varimax rotation as an exploratory method, the factor structure of an instrument to determine medical students’ perceptions of MC was investigated. Three factors were identified. The first factor comprises nine items that address attitudes toward MC, including willingness (as a future physician) to help patients access MC, obtaining training about MC in school and residency training, the physician’s role as a prescriber, cannabis having an acceptable role in medicine, efficacy of MC for chronic conditions, belief in physical health benefits of using MC, and reclassifying cannabis so that it is no longer a Schedule I drug (i.e., substances with no currently accepted medicinal use and a high potential for abuse). Concern for the potential adverse effects of MC, the second factor, consists of five items. These items address the medical student’s concern with MC’s potential for abuse/misuse, potential side effects, whether it poses serious physical and mental health risks, and its potential for addiction. Lastly, the third factor, which contains four items, addresses perceived knowledge of MC, such as confidence regarding current knowledge of MC and having knowledge about MC in general and its potential side effects.

In the area of attitudes toward MC (factor 1), two items specifically address the desire for training in MC (during undergraduate and graduate training), four items address the physician’s role and legality of prescribing MC (including medical students’ willingness to prescribe it in the future), and three items address MC’s acceptability in medical practice (including its physical health benefits and assistance for patients who suffer from chronic, debilitating medical conditions). Overall, the participants had positive attitudes regarding MC; the mean score for this factor was 2.10 (SD=0.797, range=5), where lower scores indicate more positive attitudes toward MC.

Concern for the potential adverse effects of MC (factor 2) addresses the medical student’s concern with MC’s potential for abuse/misuse and addiction (two items) and its negative effects (three items). The mean for this five-item factor was 3.39 (SD=1.101, range=5), where lower scores indicate more concern about the possible adverse effects of MC. It was conjectured that more concern about the possible negative effects of MC may be associated with the participant’s personal experience. An independent t-test was thus conducted to test for differences in concern between those participants who know of someone who used MC and who reported personal use of MC. Statistically significant differences were found; participants who knew someone who used MC reported less concern for the potential adverse effects of MC (formula). Along the same vein, participants who had personally used MC reported less concern for the potential adverse effects (formula). More research is needed to understand how nonacademic experiences with MC influence medical students’ concerns about its possible negative effects.

The third factor, perceived knowledge of MC, addresses confidence regarding current knowledge of MC (one item), having substantial knowledge about MC (one item), its potential side effects (one item), and its possible therapeutic effects (one item). The mean for this four-item factor (subscale) was 3.17 (SD=1.170, range=5). The sample consisted of 420 (79.8%) preclinical students (years 1-2) and 106 (20.2%) clinical students (years 3-4). Interestingly, only about one-fifth of the participants reported that MC is taught as part of their medical school curriculum, but the medical school from which this sample was drawn does not contain any official curricular content about MC.

Of particular interest is the perceived knowledge about MC among the participants. Slightly more than half (56.5%) agreed that they had substantial knowledge about MC, and only 47.2% felt extremely confident regarding their current knowledge of MC, yet 61% believed that they have good knowledge of the side effects of MC. Interestingly, only 22.5% of the participants reported that MC was taught as part of their medical school curriculum. While it is difficult to determine the reason for higher levels of self-perceived knowledge about MC among some students, it was thought that perhaps it was associated with the participant’s experience gained on rotations during clinical training. To further explore this phenomenon, an independent t-test was conducted to test for the differences between the preclinical- and clinical-level students; no statistically significant differences were found between the groups (p=0.076). It remains unclear why some students reported feeling more confident in their knowledge about MC and where students with higher scores on this factor obtained their information about MC, its therapeutic effects, and possible side effects.

Equally interesting, while the majority of the participants reported having positive attitudes toward MC in general (e.g., acceptable role in medicine, therapeutic value, and desire for training in MC; M=2.10), the mean scores for factor 2 (concern about the possible adverse effects of MC) were higher (M=3.39). It is important to note that the sample came from a Florida medical school. Florida is a state where qualified physicians may recommend MC for qualified patients, which could in turn influence medical students’ attitudes toward its utility and acceptance as a viable therapy.

Traditionally, MC education is not taught in medical schools in the USA and has fallen principally to physicians who opt for additional post-residency training and certification. The findings from this study indicate that medical students perceive themselves to be uninformed about MC, its effectiveness and utility, and its potentially harmful effects, as has been reported elsewhere [4,7,8,27]. While more research may be necessary, the development of curricula that incorporates MC training during the undergraduate years may be important. Medical education can play a prominent role as cannabis laws continue to change nationwide, including removing it from the list of banned controlled substances.

This brief instrument can serve as an assessment measure to evaluate the perceptions of medical students/future physicians who, as laws change and social acceptance of cannabis continues to expand, will more likely than not encounter patients who have questions about and may benefit from MC. The tool takes less than 10 minutes to complete. Individual items can be summed up for an instrument measure total, with lower total scores indicating positive attitudes, less concern for possible adverse effects, and greater perception of knowledge about MC.

Limitations

Since this sample is reflective of Florida osteopathic medical students, generalizability is limited. While allopathic and osteopathic medical training are both designed to help cure illnesses and promote health, osteopathic medical education’s focus is to train physicians to treat each patient as a whole person. Florida is one of the states in which MC can be legally prescribed and accessed by patients. To increase generalizability, a sample would be needed using both osteopathic and allopathic medical students from samples in states in which MC and/or recreational cannabis had been decriminalized. Moreover, the method used was an anonymous self-reported survey; thus, selection bias may have occurred. Due to the anonymous nature of the electronic survey response, it was impossible to ascertain if participants differed from nonparticipants.

## Conclusions

The items in the brief 18-item instrument target specific areas in which medical students maintain attitudes toward MC (including legal aspects and the role of the physician), perceive their own knowledge of MC, and express their concerns about its possible adverse effects, such as the potential for misuse/dependence and addiction. In US states where MC is not yet legal, the development of curricula that integrate MC training is encouraged as many students enter residencies in states other than those in which they went to medical school. While the measure requires further testing and development in terms of reliability and validity, it may inform future academic research about MC and provide pertinent information for medical educators to improve curricula to ensure MC readiness in their graduates.
